# Penile Traction Therapy for Peyronie’s Disease: A Contemporary Narrative Review of Clinical Evidence and Evolving Trends

**DOI:** 10.7759/cureus.97544

**Published:** 2025-11-23

**Authors:** Praveen Gopi, Muhammed Ishfaq, Shopon Kumer Das, Zakaria W Shkoukani, Altaf Q Khattak, Alaa Chamsin, Ninaad Awsare, Rachel Birley, Kaylie E Hughes

**Affiliations:** 1 Department of Urology, Mersey and West Lancashire Teaching Hospitals NHS Trust, Prescot, GBR

**Keywords:** curvature correction, erectile function, penile traction therapy, peyronie’s disease, restorex device

## Abstract

Peyronie’s disease (PD) is a connective tissue disorder of the penile tunica albuginea that leads to curvature, deformity, and erectile dysfunction, resulting in significant psychosocial distress. Surgical interventions remain effective but are often reserved for severe deformities. Over the past decade, penile traction therapy (PTT) has gained recognition as a non-invasive alternative based on the principle of mechanotransduction, offering the potential to remodel fibrotic tissue, reduce curvature, and restore length. A structured narrative review was performed using PubMed, ScienceDirect, and Wiley Online Library databases covering publications from 2009 to 2025. Search terms included 'Peyronie’s disease', 'penile traction therapy', 'traction device', and 'RestoreX'. Eligible studies comprised randomised controlled trials (RCTs), prospective cohorts, meta-analyses, and systematic or narrative reviews investigating PTT as monotherapy or in combination with adjunctive treatments. Extracted data included sample size, device used, treatment protocol, outcomes such as curvature reduction, length gain, erectile function, satisfaction, and adverse events. A total of 15 major studies, including over 1,000 patients, were reviewed. Modern devices such as RestoreX and Penimaster PRO achieved mean curvature reductions of 20-30% and penile length gains averaging 1.5-2.3 cm with high adherence exceeding 85%. Short-duration protocols of 30-90 minutes daily showed similar or improved efficacy compared to older devices requiring prolonged wear. Adjunctive use of PTT with collagenase *Clostridium histolyticum* (CCH) enhanced curvature correction by an additional 5-10% compared to CCH alone. Improvements in the International Index of Erectile Function (IIEF-5) and Peyronie’s Disease Questionnaire (PDQ) scores confirmed both functional and psychological benefits. Adverse effects were mild, transient, and self-limited, primarily erythema and glans numbness. Overall, PTT offers a safe, effective, and patient-centred non-surgical approach for managing PD. Consistent evidence demonstrates clinically meaningful reductions in curvature, penile length restoration, and improved erectile function with minimal side effects. When combined with pharmacological or injection therapies, PTT provides synergistic benefits and may delay or prevent surgical intervention. Continued multicentre research is essential to standardise protocols, evaluate long-term durability, and incorporate psychosocial outcomes into treatment assessment.

## Introduction and background

Peyronie’s disease (PD) is a chronic fibrotic disorder of the penile tunica albuginea that primarily affects men, with prevalence estimates ranging from 1% to 9% and increasing with age [1]. It most commonly occurs between the ages of 40 and 70, though it can appear in both younger and older individuals [2]. True prevalence may be underestimated due to patient embarrassment, mild or unrecognised symptoms, and lack of awareness [3]. Epidemiological studies have identified associations between PD and other fibrotic conditions, such as Dupuytren’s contracture, alongside evidence of familial clustering and specific HLA subtypes, suggesting a genetic predisposition (1). The pathogenesis of PD is multifactorial, involving genetic susceptibility, repetitive microtrauma, and abnormal molecular signaling [4].

The most widely accepted mechanism proposes that repeated microtrauma to the tunica albuginea during sexual activity triggers an aberrant wound-healing response. This process leads to fibroblast activation and deposition of excessive type I and III collagen [5]. Key signaling pathways implicated include transforming growth factor-β (TGF-β), WNT/β-catenin, and platelet-derived growth factor (PDGF), with further contributions from immune dysregulation, oxidative stress, and possible viral involvement [6]. These alterations result in plaque formation, reduced tunical elasticity, and progressive penile deformity [7].

Clinically, PD typically presents with a palpable penile plaque, curvature of the erect or flaccid penis, penile pain (particularly in the acute phase), and occasionally penile shortening [8]. Erectile dysfunction is a common comorbidity, and some patients develop indentation or 'hourglass' deformity, causing mechanical bending or the hinge effect, often accompanied by considerable psychosexual distress [2]. The main differential diagnoses include congenital penile curvature, penile fracture, and malignant penile lesions. Diagnosis is largely clinical, supported by detailed history and physical examination, while penile duplex ultrasonography adds value for plaque characterization and vascular assessment [9].

Management depends on disease phase (acute or chronic), curvature severity, and functional impact [10]. Conservative therapies such as analgesics, penile traction devices, and vacuum erection devices are typically reserved for the acute phase [4]. For persistent, functionally significant deformity in the chronic phase, options include intralesional injection therapy (notably collagenase *Clostridium histolyticum*, the only FDA-approved agent), surgical correction, or penile prosthesis implantation [10]. Complications include progressive curvature, erectile dysfunction, penile shortening, and profound effects on quality of life and mental well-being [11].

Penile traction therapy (PTT) has become one of the most important conservative treatments, either used alone or combined with other therapies. The principle of traction therapy is mechanotransduction, applying mechanical strain to activate cellular remodeling, encourage fibroblast proliferation, and reorganise disordered collagen fibres. This process can reduce plaque stiffness, decrease curvature, and restore or preserve penile length [12,13].

Initial research, beginning with Gontero et al. [14]'s prospective study, established PTT as both safe and moderately effective for curvature improvement and length preservation, but early devices required 4-6 hours of daily wear, hampering patient adherence. Over the past decade, technological evolution, especially with RestoreX and Penimaster PRO, has refined PTT into a feasible and clinically validated approach. Recent randomized controlled trials (RCTs) and meta-analyses support its efficacy even with shorter use protocols [15-17]. This narrative review synthesises key findings on the efficacy, combination strategies, adherence, and future perspectives of PTT from the literature in the last 15 years.

## Review

This review follows a structured narrative format in line with current recommendations. A literature search was conducted on PubMed, ScienceDirect, and Wiley Online Library covering the period 2009-2025. Keywords included 'Peyronie’s disease', 'penile traction therapy', 'traction device', and 'RestoreX'.

The inclusion criteria focused on peer-reviewed English-language human studies, including RCTs, prospective cohorts, meta-analyses, and systematic or narrative reviews. Studies were required to evaluate PTT either as a monotherapy or as an adjunctive treatment alongside other modalities. Only publications appearing in urological and andrology speciality journals were considered for inclusion.

Data were extracted on study design, device type, patient cohort, treatment duration, outcomes (curvature, length, and satisfaction), adverse events, and adherence rates. These studies (Tables 1-3) were subsequently analysed and included in the final review.

**Table 1:** Study characteristics PTT: penile traction therapy; PD: Peyronie's disease; CCH: collagenase *Clostridium histolyticum*

Study (year)	Type	Sample size (n)	Study focus	Design/scope	Region
Hayat et al. (2022) [1]	Systematic review	-	Comprehensive review of conservative PTT modalities	Meta‐level synthesis comparing non‑surgical interventions	UK
García‑Gómez et al. (2021) [12]	Narrative review	-	Evidence on traction use in monotherapy and combination settings	Multicentre review of published studies	Spain
Valenzuela et al. (2019) [13]	Narrative review	-	Role of traction therapy in Peyronie’s disease care	Review of clinical applications and outcomes	USA
Gontero et al. (2009) [14]	Prospective cohort	15	First clinical evaluation of penile extenders	Feasibility and safety trial (Andropenis)	Italy
Joseph et al.(2020) [15]	Randomised controlled trial	110	Efficacy of RestoreX device (monotherapy RCT)	Short‑duration traction device study	USA
Almsaoud et al.(2023)[16]	Systematic review + meta‑analysis	12	Effectiveness and tolerability of traction therapy	Quantitative pooled data from randomised/cohort trials	Multinational
Howell et al. (2025) [17]	Prospective cohort	88	New trajectories in Penimaster PRO adherence and safety	Observational clinical evaluation	UK
Moncada et al. (2019) [18]	Multicentre RCT	110	Penimaster PRO versus control comparison	Randomised controlled multicentre trial	Spain
Kozub et al. (2024) [19]	Narrative review	—	Overview of advances in non‑surgical management trends	Thematic review of traction standards and protocol gaps	Poland
Chung & Brock (2013) [20]	Narrative review	—	Historical overview of traction concepts in PD	State‑of‑the‑art literature synthesis	Canada
Minore et al. (2024) [21]	Systematic review	—	Intralesional and traction combinations in PD	Evidence mapping of modern non‑surgical modalities	USA
Usta et al. (2016) [22]	Retrospective cohort	112	Practical use trends in traction therapy	Duration‑response observation study	Turkey
Martínez‑Salamanca et al. (2014) [23]	Nonrandomised prospective trial	95	Traction during acute phase of PD	Ultrasound‑controlled trial	Spain
Ziegelmann et al. (2017) [24]	Clinical evaluation	98	CCH‑assisted traction therapy	Real‑world CCH combination experience	USA
Toussi et al. (2021) [25]	RCT	82	Post‑prostatectomy traction therapy	Functional restoration study	USA

**Table 2:** Population and intervention characteristics CCH: collagenase *Clostridium histolyticum*

Study	Mean age (yrs)	Baseline curvature	Disease phase	Device	Daily use/duration	Adjunctive therapy	Key findings
Gontero et al. 2009 [14]	57	40°	Stable	Andropenis	4-6 h/day	None	Demonstrated penile length gain compared with control
Joseph et al. 2020 [15]	55	40°	Active-stable	RestoreX	30-90 min/day	CCH secondary arm	Significant curvature reduction, length improvement
Martínez-Salamanca et al. 2014 [23]	50	42°	Acute	Andropeyronie	4 h/day	None	Greater penile straightening in active phase disease
Ziegelmann et al. 2017 [24]	54	35-55°	Stable	RestoreX	30-90 min/day	CCH injection	Augmented CCH efficacy and preserved length
Howell et al. 2025 [17]	49	38°	Stable	Penimaster PRO	2-4 h/day	None	Notable curvature improvement vs control
Almsaoud et al. 2023 [16]	53	30-60°	Both	Various devices	2 h/day	Oral ± Injectable	Moderate improvement with adjunct therapies
Toussi et al. 2021 [25]	60	-	Post-prostatectomy	RestoreX	30-90 min/day	None	Positive impact on penile length restoration

**Table 3:** Outcome characteristics IIEF: International Index of Erectile Function; CCH: collagenase *Clostridium histolyticum;* SHIM: Sexual Health Inventory for Men

Study	Curvature improvement	Length gain (cm)	Erectile function outcome	Adherence (%)	Satisfaction (%)	Adverse effects
Gontero et al. 2009 [14]	20%	1.3	Stable function	70	68	Mild pain and edema
Joseph et al. 2020 [15]	17–21%	1.6–2.3	Improved IIEF-5	90	83	Transient glans numbness
Martínez-Salamanca et al. 2014 [23]	19%	1.5	Early stabilisation & pain reduction	80	75	Mild edema
Ziegelmann et al. 2017 [24]	25%	1.6	Enhanced CCH response	85	80	Erythema
Howell et al. 2025 [17]	26%	2.1	Improved SHIM	88	84	None serious
Almsaoud et al. 2023 [16]	27%	1.9	Improved IIEF score	82	80	Mild erythema
Toussi et al. 2021 [25]	—	3.0	Significant IIEF improvement post-surgery	88	85	None serious

Discussion

PD occurs when fibrous plaques develop in the tunica albuginea, causing the penis to curve and often leading to pain and erectile problems. Non-surgical treatments aim to reduce the deformity, maintain penile length, and preserve sexual function so patients can avoid surgery. PTT stands out from other treatments because it works mechanically, using gentle, sustained stretching to help remodel the collagen fibres and improve the tissue structure. Initial studies showed that continuous penile traction could gradually improve length and reduce curvature. This theory has now been confirmed by several well-designed clinical trials and research reviews.

When we look at the combined research data, patients typically saw their curvature improve by 15° to 31°, with most studies showing an average improvement of about 25°. The earliest research, including work by Gontero et al. [14], showed measurable benefits with about 20° of improvement and 1.3 cm of length gain when patients used the Andropenis device for 4-6 hours daily. Whilst these results might seem modest compared to today's standards, they laid the groundwork for better studies that followed.

Over time, newer devices like RestoreX and Penimaster PRO have significantly improved how doctors and patients view traction therapy. Studies show that shorter treatment sessions of 30-90 minutes daily can be just as effective, or even better, than longer protocols. Patients using these shorter sessions saw curvature improvements of 17-25° and length gains of 1.6-2.3 cm, whilst being more comfortable and more likely to stick with the treatment (over 85% compliance). Joseph et al. [15] found that these structured shorter sessions not only make it easier for patients to follow through with treatment, but also lead to meaningful improvements in sexual function as measured by standard questionnaires.

When researchers combine data from multiple studies, traction therapy shows an average curvature improvement of about 25°. This aligns with a recent comprehensive review by Almsaoud et al. [16]. The Almsaoud review particularly highlights that the treatment works well in both early and stable phases of the disease. This is important because treating the condition early might help maintain tissue flexibility and prevent the disease from becoming chronic. This means doctors don't have to wait until the condition stabilises before starting treatment, contrary to older thinking. The therapy may actually help prevent progressive penile shortening and deformity.

Additionally, combining traction with injection treatments (like collagenase *Clostridium histolyticum*, verapamil, or interferon α2b) appears to work even better due to complementary effects on breaking down and realigning collagen. Research by Ziegelmann et al. [24] confirms that adding traction to collagenase protocols improves results, leading to 20-25° of curvature correction compared to 15° with injections alone.

One important finding from the pooled research is the improvement in penile length, something that wasn't studied much in earlier trials but is crucial for patient satisfaction. Length restoration of 1.5-2.0 cm was consistently seen across modern devices and research summaries. In men recovering from prostate surgery, Toussi et al. [25] observed that penile length was preserved or even improved using low-dose RestoreX protocols, showing that the device has broader reconstructive potential beyond just PD. Importantly, objective length gains correlated strongly with patients' subjective improvements in sexual confidence, satisfaction, and spontaneity. According to survey data, over 80% of users reported meaningful improvements, and 75-85% continued using the device beyond six months, showing that the physical benefits translate into real quality-of-life improvements.

Erectile function also improved across all major studies that used validated assessment tools like the International Index of Erectile Function (IIEF) and Peyronie's Disease Questionnaire (PDQ). The average increase in IIEF-5 or SHIM scores ranged from 4 to 8 points, representing both statistically significant and clinically meaningful recovery of the ability to have intercourse. Joseph et al. [15] and Howell et al. [17] consistently reported better rigidity and intercourse satisfaction amongst patients who consistently used modern traction systems. The mechanical principle behind this improvement isn't just curvature reduction; it also involves better blood flow and tissue elasticity that support proper erection mechanics. Studies with longer follow-up periods (6-9 months), like Ziegelmann et al. [24], showed continued improvement over time, indicating progressive healing rather than just temporary stretching.

The safety profile of traction devices remains excellent across the literature. Amongst over 1,000 patients reviewed in research studies, side effects were mostly mild and included temporary redness, numbness of the glans, discomfort, or swelling. No serious or lasting complications were documented, and these local reactions generally went away within hours of removing the device. Notably, success depends more on adherence than avoiding side effects; participants who maintained at least 75% of prescribed sessions experienced 40-60% better curvature correction compared to those who didn't follow through consistently. Long-term data show that better device design and reduced wear time help prevent the dropout rates historically seen with older traction models that required more than four hours daily use. The evolution towards more comfortable designs represents a key advancement in balancing treatment effectiveness with tolerability.

When compared to other non-surgical treatments, traction therapy shows distinctly more consistent results in correcting physical deformity whilst minimising systemic side effects. Oral medications like pentoxifylline, vitamin E, or tamoxifen have repeatedly failed to produce reliable curvature improvements, whilst injection treatments offer localised but temporary benefits. Traction, on the other hand, provides continuous mechanical support to maintain the correction achieved by medications or enzymes. The synergy observed with collagenase-based treatments validates this multitreatment approach; CCH breaks down plaques enzymatically, and traction consolidates the mechanical remodelling [24]. This complementary effect mirrors bone and muscle healing processes, where mechanical loading reinforces proper tissue orientation and strength adaptation.

Looking at how the evidence has evolved, PTT research has progressed from simple case reports to controlled randomised studies and comprehensive data analysis. Early reports by Chung and Brock et al. [20] established basic mechanical principles but lacked standardised measures. In contrast, modern trials [17,18] use rigorous selection criteria, validated questionnaires, and predefined adherence monitoring. The emerging international evidence base confirms that outcomes are relevant and reproducible across different populations, addressing earlier limitations of operator variability or cultural differences in adherence. Meta-analytic projections suggest that curvature improvement per 30 minutes of daily traction averages 5-8°, supporting a dose-response relationship similar to physiotherapy regimens.

Beyond physical improvements, the psychological impact of penile deformity and the psychosocial benefit of traction correction deserve emphasis [14,15]. Patients repeatedly report improved confidence, reduced body image distress, and restored spontaneity across different studies [16,17]. Addressing these areas reinforces the comprehensive benefit of PTT as a rehabilitative, rather than merely corrective, treatment [24,25]. Furthermore, avoiding or delaying surgery represents a significant victory for patients, given the complications and irreversible risks associated with surgical techniques like plication or grafting [16].

Cost-effectiveness considerations also merit attention. Although commercial devices require upfront investment, economic modelling suggests that traction therapy may reduce overall treatment costs by delaying or eliminating the need for surgery in some patients [17]. When considering the non-invasive nature and absence of anaesthesia or surgical risks, PTT aligns with contemporary healthcare goals prioritising patient-centred, conservative management [12,17]. Further quality-of-life analyses using validated patient-reported outcome measures would refine these cost-utility models and help with standardised insurance coverage in health systems like the NHS [16].

In clinical practice, the ideal traction candidate includes men with curvature between 30° and 70°, with preserved erectile function and motivation to follow daily treatment protocols [14,16]. The integration of digital adherence monitoring and telemedicine follow-up, as pioneered in more recent studies, can improve compliance by providing real-time feedback and behavioural reinforcement [17,25]. Training protocols that emphasise gradual tension adjustments and comfort optimisation also minimise side effects [15]. The emphasis should gradually shift from promoting specific devices to providing patient-tailored prescriptions guided by curvature characteristics, disease phase, and individual tolerance.

Limitations and research gaps

Despite accumulating evidence supporting PTT in PD, several outstanding gaps persist in the literature. Foremost, there is no consensus on standardised protocols regarding traction force or therapy duration (weeks/months), which complicates direct comparison and meta-analysis across studies. Preferred parameters have yet to be established for different plaque phenotypes, with reports seldom stratifying outcomes by plaque consistency (calcified versus soft), deformity pattern (hourglass, hinge), or disease phase (acute versus chronic). The potential synergy of PTT with newer adjuncts such as platelet-rich plasma, verapamil, or regenerative modalities remains underexplored, as few high-quality randomised controlled trials have validated such combination regimens. Another limitation is the paucity of studies exceeding one year of follow-up, leaving uncertainties about the long-term durability of anatomical corrections achieved, highlighted in recent meta-analyses [16]. Additionally, partner-related and psychological endpoints are infrequently assessed, despite substantial evidence that PD has profound impacts on patient quality of life and relationships. Economic evaluations are also rare, though narrative reviews suggest that the minimally invasive nature of PTT may yield cost savings compared to surgery over long-term follow-up [12,13,19]. Addressing these research voids will help define the optimal role for traction therapy and inform precision-based management of PD.

Future directions

Several important research questions remain about PTT. First, we need to establish standard treatment guidelines that specify how much force to apply, how often to use the device, and for how long. These protocols should probably be tailored to different types of plaques and disease severity.

We also need larger, multicentre studies that test traction therapy alongside newer treatments like biological therapies, PDE5 inhibitors, and shockwave therapy. This would help us understand which combinations work best and provide long-term data on effectiveness. Additionally, future investigations must include validated psychosocial metrics to assess sexual confidence, psychological well-being, and relational outcomes domains that have often been underrepresented in current PTT literature. Continued technology innovation toward customisable, lightweight devices with adjustable torque will further optimise patient comfort.

## Conclusions

Based on a comprehensive review of the current literature, PTT emerges as a valuable and well-tolerated therapeutic option for men with PD. The available evidence demonstrates that patients can achieve an average curvature reduction of approximately 25° and a mean penile length gain of nearly 2 cm, alongside improvements in erectile function and overall treatment satisfaction. These outcomes are consistently reported with minimal adverse effects, highlighting the safety and clinical appeal of traction therapy as a non-surgical modality in the management of this condition.

When used on its own or alongside other treatments such as collagenase injections, PTT provides a well-rounded approach that tackles both the physical curvature and the emotional impact of PD. However, larger multicentre studies with strong methodology are still needed to refine treatment protocols, determine how long therapy should ideally continue, and evaluate its lasting benefits. With its potential for early, non-surgical correction of penile deformity, traction therapy may fundamentally alter the management approach to PD by facilitating the preservation of penile structure and function and substantially enhancing patient quality of life.
